# Transmission studies of the newly described apple chlorotic fruit spot viroid using a combined RT-qPCR and droplet digital PCR approach

**DOI:** 10.1007/s00705-020-04704-5

**Published:** 2020-07-08

**Authors:** Thomas Leichtfried, Helga Reisenzein, Siegrid Steinkellner, Richard A. Gottsberger

**Affiliations:** 1 Institute for Sustainable Plant Protection, Austrian Agency for Health and Food Safety, 1220 Vienna, Austria; 2 Institute of Plant Protection, University of Natural Resources and Life Sciences, 3430 Tulln an der Donau, Austria

## Abstract

The transmission of the apscaviroid tentatively named apple chlorotic fruit spot viroid (ACFSVd) was investigated using a one-step reverse-transcription (RT) droplet digital PCR assay for absolute quantification of the viroid, followed by quantification of relative standard curves by RT-qPCR. Our results indicate that ACFSVd is effectively transmitted by grafting, budding and seeds. No transmission has yet been observed to the viroid-inoculated pome fruit species *Pyrus* sp. and *Cydonia* sp. ACFSVd was detected in viruliferous aphids (*Myzus persicae*, *Dysaphis plantaginea*) and in codling moths (*Cydia pomonella*). The viroid was also detected systemically in the infected hemiparasitic plant *Viscum album* subsp. *album* (mistletoe).

**Electronic supplementary material:**

The online version of this article (10.1007/s00705-020-04704-5) contains supplementary material, which is available to authorized users.

Apple chlorotic fruit spot viroid (ACFSVd) is a putative new pathogenic viroid on apple that was recently detected in the Austrian province of Burgenland. This viroid causes chlorotic fruit spots and bump-like symptoms on the skin of apples, thus reducing fruit quality and making the fruits unmarketable [1]. It is crucial that key epidemiological facts be understood to reduce the risk of the spread of this putative new pathogen. For viroids, several means of dispersal are known, such as horizontal transmission by pruning shears, grafting knives or machinery, or vertical transmission by seeds and pollen [2–5]. Additionally, the international trade of propagative material can play a key role in long-distance transmission, because viroids infect plants systemically [6, 7]. Vector-based transmission may have a significant influence on the spread of diseases. Important vectors of viroids are green peach aphids (*Myzus persicae)* and white flies (*Trialeurodes vaporariorum*) [8–12] as well as bumblebees [4, 13]. Further unobserved modes of transmission could involve harmful insects (e.g., codling moths) and hemiparasitic plants such as mistletoe (*Viscum album* subsp. *album* L.), which is frequently found in extensively managed apple orchards. These plants come directly into contact with host plants through haustoria [14] and can apparently take up genetic material such as RNA [15]. For the detection and characterization of viroids, various techniques can be used, such as biological indexing [16], PCR-based methods [17–19], and next-generation sequencing [1, 20, 21]. A qualitative result can be obtained with endpoint RT-PCR, and relative quantification can be achieved by RT-qPCR. For quantification with qPCR, a standard curve with known concentrations of the target is necessary to transform the qPCR output of the quantification cycle (Cq) into absolute concentrations [22, 23]. For culturable microorganisms such as bacteria, cell suspensions with defined concentrations can be prepared from cultures [24]. In contrast, viroids are obligate cell parasites [25], and therefore, it is not possible to define an absolute target concentration of viroid per sample using classical molecular methods. For absolute quantification, digital PCR (dPCR) is a helpful tool for determining the exact number of target copies. One of the main advantages of dPCR is absolute target quantification without reference to a calibration curve [26]. There are different platforms available, which can be distinguished based on their partitions (chambers or droplets) [27]. In this study, droplet digital PCR (ddPCR) was used for the absolute quantification of ACFSVd. This technology has already been used for plant pathogens such as phytoplasma [28], *Erwinia amylovora*, *Ralstonia solanacearum* [29], *Xylella fastidiosa* [30] potato virus Y [22], citrus yellow vein clearing virus [31] and citrus tristeza virus [32]. To our knowledge, there is no published report on the use of ddPCR for the quantification of pathogenic viroids in plant tissue. In this study, we investigated the mode of transmission of ACFSVd. We addressed transmission by insects, sap inoculation, infected scions, and seeds. As we found heavy infection in mistletoe on ACFSVd-symptomatic apple trees of cultivar “Ilzer Rose” at the site where this viroid was first described, we also included this possibly new pathway of viroid transmission in this study. For analysis of ACFSVd transmission, we developed a new specific RT-qPCR assay, which was combined with an RT- ddPCR assay for absolute quantification of this viroid.

For RT-qPCR and ddPCR, plant material infected with the ACFSVd isolate (GenBank no. MF521431.2) was collected from symptomatic apple fruit of the local cv. “Ilzer Rose” in the Austrian province of Burgenland. Total RNA was extracted directly from symptomatic apple fruit skins using a Spectrum Plant Total RNA Kit (Sigma Aldrich, St. Louis, USA), following the manufacturer’s instructions. The RNA was eluted in 50 µl of elution solution, which was provided in the kit and stored at -20 °C until use. For absolute quantification by ddPCR, a 1:1,000 dilution was prepared.

Twenty seeds were extracted from ACFSVd-symptomatic apple fruit. For testing the cotyledons, the seed coats of 25 seeds were excised using sterile scalpel blades. Fifty aphids feeding on symptomatic trees and five codling moth larvae feeding on symptomatic apple fruits were collected. The biological materials were frozen at -80 °C for 30 min. Seeds, cotyledons and insects were put into a 2-ml Lysing Matrix A tube (MP Biomedical, California, USA) and crushed in a FastPrep-24 instrument (MP Biomedical, California, USA) for 30 s at 6.5 m/s. The entire smashed sample was used for total RNA extraction using an RNeasy Plant Mini Kit (QIAGEN, Hilden, Germany), following the manufacturer’s instructions. The same commercial kit was used to extract RNA from 100 mg of plant material from twigs, leaves, and 20 buds from symptomatic trees. For the transmission experiments ACFSVd-infected scions were grafted onto two-year-old apple (*Malus sylvestris*), pear (*Pyrus communis*), and quince (*Cydonia oblonga*) trees.

A total of 150 seeds of mistletoe grown on symptomatic apple trees were tested. For this purpose, a phenol:chloroform:isoamyl alcohol extraction as described by Psifidi et al. [33] was used with minor modifications. Mistletoe seeds are surrounded by a viscous substance containing viscin [34]. Due to this sticky substance, a commercial kit could not be used, because viscin causes the liquid to become viscous during the extraction steps, impeding elution, e.g., through silica columns. To separate the seeds from the viscous arillus, the samples were incubated at 30 °C for 24 h. After the arillus was dried, it could be almost completely removed mechanically from the seeds by scraping it off with sterile scalpel blades. One seed was added to a Lysing Matrix A tube prefilled with 500 µl NTES buffer (1 M NaCl, 0.5% SDS, 10 mM, Tris-HCl, pH 8.0, 1 mM EDTA) and 500 µl of phenol:chloroform:isoamyl alcohol solution (25:24:1) (Applichem, Darmstadt, Germany). The seeds were comminuted using a FastPrep-24 instrument as mentioned above. After a centrifugation step (30 min at 16,000 *g*, 8 °C), the upper aqueous phase was transferred into a new 1.5-ml tube. Then, 500 µl of chloroform:isoamyl alcohol (VWR, Pennsylvania, USA) was added to the tube, which was then vortexed and centrifuged at 16,000 *g* for 10 min. The aqueous phase was again pipetted into a new 1.5-ml tube, and the nucleic acid was precipitated by adding 1.5 volumes of ice-cold isoamyl alcohol (Merck, Darmstadt, Germany) and 0.1 volumes of sodium acetate (0.3 M) and keeping the sample in a freezer for 12 h. After a centrifugation step (16,000 *g*, 30 min), the supernatant was discarded. The pellet was washed with 200 µl of 70% ethanol (Scharlab S.L., Barcelona, Spain) and centrifuged for 5 min at 5200 *g*. The supernatant was discarded, and this step was repeated. The pellet was vacuum dried using a vacuum concentrator (Eppendorf, Hamburg, Germany) and dissolved in 40 µl of PCR-grade water. Seed transmission was tested as described by Kim et al. [35]. For germination, 144 seeds from symptomatic apples were kept in a refrigerator for 60 days to break seed dormancy. The seeds were planted in pots, and seedlings were grown in a glasshouse. After two months, the plantlets were homogenized using a Homex leafpress (Bioreba AG, Reinach, Switzerland) in Bioreba bags, and an aliquot of the homogenate was used for extraction of total RNA using an RNeasy Plant Mini Kit (QIAGEN, Hilden, Germany) following the manufacturer’s instructions. Primers ACFSVd-Frt (5′-TTAGGACCGCGGAGCTGTTG-3′) and ACFSVd-Rrt (5′-ACGAGTCCCTCGACCCTCT-3′) were designed by aligning the full ACFSVd sequence (GenBank no. MF521431.2) obtained from the National Center for Biotechnology (NCBI) using the Primer BLAST tool (Software Primer 3 and BLAST) [36]. The specificity of the newly designed primer for the annealing site was tested *in silico* at NCBI GenBank and *in vitro* using 5x HOT FIREPol® EvaGreen® qPCR Supermix (Solis Biodyne, Tartu, Estonia). After *in vitro* specificity testing with ACFSVd and other related viroids (Supplementary Table S1), a FAM TaqMan minor groove binder (MGB) nonfluorescent quencher hydrolysis probe (ACFSVd-P 5′-FAM-GTTCCTGTGGTGACACCTCC-MBGEQ-3´) was synthesized at Eurofins Genomics (Cologne, Germany). The reaction was performed in a 10-µl final volume containing, 5 µl of 2x qScript XLT 1-Step RT-qPCR ToughMix (QuantaBio, Beverly, USA), 0.5 µl of 10 µM forward and reverse primer, 2 µl of 1 µM probe, 1 µl of water and 1 µl of template. PCR was performed in a magnetic-induction cycler (Bio Molecular Systems, Australia) under the following conditions: reverse transcription at 49 °C for 30 min, denaturation at 95 °C for 5 min, and 40 cycles of 95 °C for 15 s and 60 °C for 60 s. The standards for the relative quantification were prepared from the absolutely quantified RNA determined by ddPCR. The standard was serially diluted tenfold down to standard 5 (13.4 copies/µl), and standard 6 was then made by diluting standard 5 1:4, with a resulting copy number of 3.35. The purpose of the deviating dilution of standard 6 was to obtain a better resolution of the standard curve. For all standards, three technical replicates were used (Fig. 2). ddPCR was carried out on RNA extracted from symptomatic Ilzer Rose apple fruit skin according to the manufacturer’s recommendations [37]. The reaction was prepared using a One-Step RT-ddPCR Advanced Kit (Bio-Rad, USA) in a 20-µl final volume with the following components: 5 µl of Supermix, 2 µl of reverse transcriptase, 1 µl of 300 mM dithiothreitol (DDT), 1.8 µl of 10 µM forward and reverse primer, 0.5 µl of 10 µM probe, 6.9 µl of water and 1 µl of template. The same primers and probe used in the RT-qPCR were used for the RT-ddPCR. The PCR was performed in a VWR PCR Thermal Cycler XT^96^ Gradient (VWR, USA) at a ramping rate of 2.5 °C/s. The cycling conditions were adapted to the manufacturer’s protocol [37]. For the optimization of the RT-ddPCR reaction, different annealing temperatures (60-55 °C) and primer and probe concentrations were used (data not shown). For absolute quantification, the QX200 droplet reader was used with QuantaSoft software version 1.7.4.0971 (Bio-Rad, USA) for droplet counting. More than 10,000 accepted droplets were required for each well for them to be used for further processing [23].

After testing of viroids of different species for specificity (Supplementary Table S1) and *in silico* comparison of primers and probes with sequences from the NCBI GenBank database, the assay proved to be 100% specific for ACFSVd. The expected fragment size was calculated to be 128 bp, and this was confirmed using a QIAxcel capillary electrophoresis system (QIAGEN, Hilden, Germany) and a screening cartridge with standard settings (data not shown). The analytical sensitivity of the assay was determined by preparing a standard curve using three replicates of a dilution series. For the correlation with absolute copy numbers, the same sample used in the ddPCR was used for the dilution series (Fig. 2) and was detected in three replicates down to a calculated concentration of 3 viroid molecules per µl. The dilution with 3 viroid RNA molecules was always amplified by the assay, with cycle threshold (C_t_) values of approximately 36 (Fig. 2). The efficiency of the assay could be shown by analysing the standard curve [24]. The equation was y = -3.05x + 36.23, and the calculated efficiency was 1.13 (113%). The correlation coefficient of the standard curves (*r*-squared) was 0.98. The tested samples were all set in relation to the standard curve.

Different primers, probe concentrations, and annealing temperatures were tested to optimize the resolution of positive and negative clustered droplets. The best droplet resolution was achieved by using a 1:1,000 dilution of the RNA extracted from symptomatic apple fruit skin, 900 nM primers, 250 nM probe, and an annealing temperature of 56 °C. The threshold for droplet positivity was set manually at horizontal line 2,674 and Ch1 amplitude (Fig. 1). The mean concentration of detected ACFSVd target copies (three technical replicates) in the material tested from 100 mg of symptomatic apple fruit skins was 1,336,000 copies/µl (calculated value) ± 54,809 (SD). The average number of targets per droplet (λ-value) was calculated using the following equation: λ = − ln × (1 − k∕n) [38]. From three technical replicates, the range of accepted droplets was 12,683 to 15,747. The range of positive droplets was 690 to 842, and the range of the λ-value was 0.05495 to 0.05942.

**Fig. 1:**
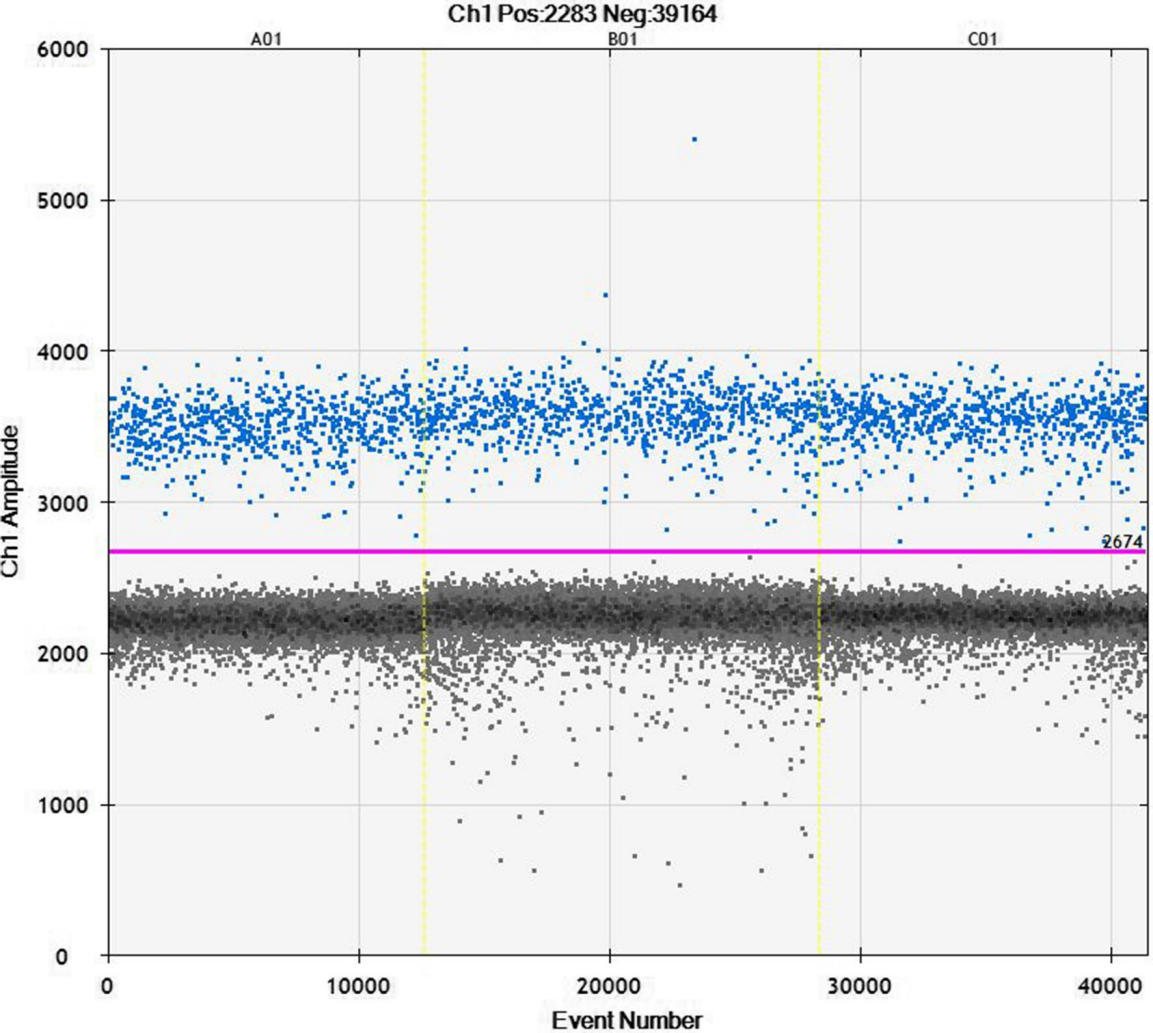
1D amplitude view of a ddPCR ACFSVd run. The best cluster of positive (upper cluster) and negative (lower cluster) droplets was observed when using 900 nM primers and 250 nM probe

**Fig. 2:**
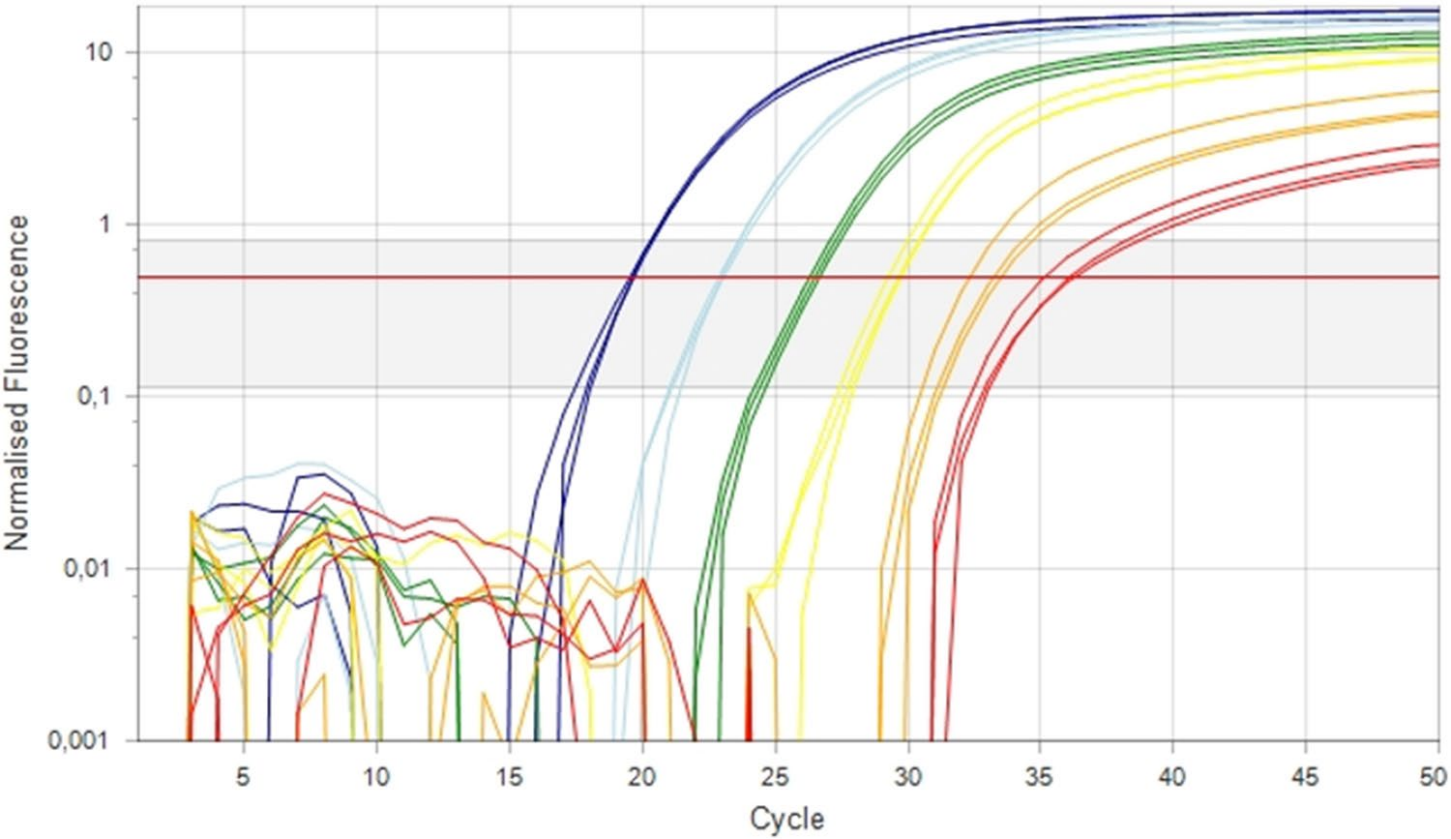
Standard curve and fluorescence amplification plot of six standards (dilutions) and three technical replicates. The ACFSVd standard curves from 1,336 copies × 10^5^/µl to 3.35 copies/µl in three replicates (left to right) were drawn using MIC software (v 2.6.4)

ACFSVd could successfully be transmitted to healthy apple trees by top grafting and budding [1]. Transmission to *Pyrus* sp. or *Cydonia* sp. could not yet be confirmed by RT-qPCR.

ACFSVd was detected in apple seeds and in dissected embryos (Table 1). Seedlings that germinated from infected apple seeds showed an infection rate of 2.8%. Furthermore, the viroid was detected in aphids (*M. persicae*, *D. plantaginea*) and in larvae of *C. pomonella* that had been feeding directly on symptomatic apples. ACFSVd was identified in the leaves, stems and seeds (without the arillus) of *V*. *album* subsp*. album*. The amount of ACFSVd copies/sample detected in *C. pomonella* larvae extracted from symptomatic fruits ranged from 1.48 × 10^5^ to 1.54 × 10^5^. Germinated seedlings showed an infection rate of 2.8%. The viroid titer of 30 to 400 copies/seed was low. ACFSVd could also be detected in aphids with a viroid titer of 100 to 800 copies/insect. Increased amounts of viroid were found in the plant materials and buds of symptomatic trees. (Table 1).

**Table 1:** Results obtained with different samples and determination of target copies/sample quantified by RT-qPCR

Sample material	ACFSVd RNA copies/sample (minimum)	ACFSVd RNA copies/sample (maximum)
Seeds from symptomatic apple fruit	6,000	220,000
Cotyledon from symptomatic apple fruit	2,000	500,000
Seedling from symptomatic apple fruit	30	400
Seeds (*V*. *album* subsp. *album*)	50	1,900
Plant material (100 mg of *V*. *album* subsp. *album*)	200	1.200
Plant material (100 mg of phloem and leaves) from symptomatic apple trees	1.5 × 10^8^	1.2 × 10^9^
One bud from a symptomatic apple tree	3.000	7.7 × 10^6^
Aphids (*M. persicae*, *D. plantaginea*) from a symptomatic apple tree	100	800
Larvae of *C. pomonella* from symptomatic apple fruit	148,000	154,000

In this study, RT-qPCR and RT-ddPCR were used to detect ACFSVd in a transmission study based on absolute quantification of the viroid. ACFSVd consists of a small RNA molecule of 354 nt [1]. An RT-qPCR assay was developed to detect and quantify ACFSVd in infected plant tissues and viruliferous insects. For relative quantification, standard curves were used for qPCR [39]. Here, we combined ddPCR and standard curves generated by RT-qPCR for relative quantification of the viroid based on absolute quantities (target copies). The main advantage of this new application of ddPCR and RT-qPCR is that the absolute quantification has to be done only for the positive control and then can be used for defined standard curves in qPCR. These defined standard curves can be applied to determine the detection sensitivity of qPCR assays, set detection limits used in quality management, and determine transmission rates. Furthermore, this method is more cost-effective and time-saving than determining the absolute target copies for each sample.

In our study, the viroid titer in buds and twigs from symptomatic apples was high. Our results support the assumption that ACFSVd could be transmitted very efficiently by horizontal transmission, such as via top-grafting, budding, and propagative material. Vertical transmission was confirmed by testing germinated seedlings. The infection rate of 2.8% was slightly lower than that of apple scar skin viroid (ASSVd) (7.7%) [35]. The low infection (or seed transmission rate) could be explained by the generally low viroid titer of 30 to 400 copies/seed. To our knowledge, vertical transmission has only been reported for the previously mentioned viroids in the genus *Apscaviroid*. In our study, ACFSVd was also detected in dissected cotyledons of infected apple seeds. The number of ACFSVd molecules was similar in seeds and cotyledons, indicating that ACFSVd is mostly located in the embryo rather than in the seed coat. Horizontal transmission was confirmed by transmission via budding from infected plants to apple trees of the cvs. Topaz and Gala and the rootstock M9. However, no symptoms on apple fruit could be recorded because the trees were not yet in a generative phase. Transmission by grafting to other pome fruits such as, *P. communis* and *C. oblonga* could not be confirmed. Our tests will be ongoing for the next few years to clarify the susceptibility of members of taxa other than *Malus* sp. to ACFSVd. To date, no apscaviroid transmission to pome fruits by insects has been reported [40–42], while one instance of transmission to plants other than pome fruits has been reported. Walia et al. [8] reported the transmission of ASSVd from infected cucumber and bean plants to several herbaceous plant species by *Trialeurodes vaporariorum*. We found that the titer of ACFSVd in feeding insects is generally low. However, this may be the reason why apscaviroids are not readily transmitted by feeding insects. Nielsen et al. [43] demonstrated that insects with a low titer of potato spindle tuber viroid were not able to transmit the viroid under experimental conditions, concluding that a larger number of target copies is required for viroids to be effectively transmitted to other plants or hosts. In our study, the ACFSVd titer in *C. pomonella* larvae was significantly higher than that in aphids. Further research must be carried out to clarify whether this insect might be a new potential vector of viroids to pome fruits. Another possible new pathway of ACFSVd transmission is by mistletoes. These hemiparasitic plants are in contact with their hosts, such as apples and other Rosaceae species [44], by haustoria [14]. In this way, mistletoes absorb water and nutrients [45–47]. For another parasitic plant, *Cuscuta* spp., it is known that haustoria also transfer macromolecules [48], mRNAs [49], metabolites [50], and pathogens such as viroids [51], viruses [52] and phytoplasmas [53]. Thus, an exchange of ACFSVd RNA molecules between mistletoes and their host plants has to be considered. Moreover, some bird species feeding on seeds of mistletoe are able to distribute the seeds over a long distance [54, 55], and ACFSVd-infected mistletoe seeds could also be spread in this way. For the seed testing of *V*. *album* subsp. *album*, an RNA extraction procedure was adapted, optimized, and used successfully for these viscin-containing samples. However, in our study, we detected ACFSVd not only in seeds but also in all other parts of the mistletoe. In particular, the spread of infected mistletoe seeds could be a so far undiscovered pathway for the distribution of plant-pathogenic viroids in tree hosts. In summary, we show that ACFSVd is transmitted by top grafting and budding. The viroid is seed-borne, seed-transmitted, and detected in viruliferous aphids and codling moths as well as infected mistletoe seeds.

## Electronic supplementary material

Below is the link to the electronic supplementary material.

Supplementary material 1 (DOC 43 kb)
