# Correction to: Transmission studies of the newly described apple chlorotic fruit spot viroid using a combined RT-qPCR and droplet digital PCR approach

**DOI:** 10.1007/s00705-020-04753-w

**Published:** 2020-08-30

**Authors:** Thomas Leichtfried, Helga Reisenzein, Siegrid Steinkellner, Richard A. Gottsberger

**Affiliations:** 1 Institute for Sustainable Plant Protection, Austrian Agency for Health and Food Safety, 1220 Vienna, Austria; 2 Institute of Plant Protection, University of Natural Resources and Life Sciences, 3430 Tulln an der Donau, Austria

## Correction to: Archives of Virology 10.1007/s00705-020-04704-5

Authors would like to update the given name and family name of authors which were incorrect in the original version.

The original article has been corrected.

