# Supplementary material for: Transmission studies of the newly described apple chlorotic fruit spot viroid using a combined RT-qPCR and droplet digital PCR approach
Source: Arch Virol. 2020 Jul 8;165(11):2665–71. doi: 10.1007/s00705-020-04704-5 (PMC7547948; doi:10.1007/s00705-020-04704-5)
Supplement: Supplementary file 1 — Supplementary material 1 (DOC 43 kb) [file 705_2020_4704_MOESM1_ESM.doc]

**Supplementary Table S1:** Specificity test with various viroid species and qualitative result of the RT-qPCR

| **Abbrevation** | **Viroid species** | **Host plant** | **Geographical origin** | **RT-qPCR results** |
| --- | --- | --- | --- | --- |
| **ACFSVd** | Apple chlorotic fruit spot viroid | *Malus* *domestica* | Austria | + |
| **ASSVd** | Apple scar skin viroid | *Malus* *domestica* | Italy | - |
| **HSVd** | Hop stunt viroid | *Citrus* sp. | Italy | - |
| **HSVd** | Hop stunt viroid | *Citrus* sp. | Malta | - |
| **PBCVd** | Pear blister canker viroid | *Pyrus* *communis* | Italy | - |
| **PBCVd** | Pear blister canker viroid | *Pyrus* *communis* | Austria (Burgenland) | - |
| **PBCVd** | Pear blister canker viroid | *Pyrus* *communis* | Austria (Styria) | - |
| **PLMVd** | Peach latent mosaic viroid | *Prunus* *persica* | Italy | - |
| **CDVd** | Citrus dwarfing viroid | *Citrus* sp. | Malta | - |
| **HLVd** | Hop latent viroid | *Citrus* sp. | Slovenia | - |
| **CBCVd** | Citrus bark cracking viroid | *Citrus* sp. | Slovenia | - |
| **TASVd** | Tomato apical stunt viroid | *Solanum* *jasminoides* | Austria | - |
| **PSTVd** | Potato spindle tuber viroid | *Capsicum* *anuum* | Austria | - |
| **CEVd** | Citrus exocortis viroid | *Citrus* sp. | Malta | - |
